# Loss of Asthma Control in Pediatric Patients after Discontinuation of Long-Acting Beta-Agonists

**DOI:** 10.1155/2012/894063

**Published:** 2012-08-23

**Authors:** Adrian R. O'Hagan, Ronald Morton, Nemr Eid

**Affiliations:** Division of Pediatric Pulmonology, University of Louisville, Louisville, KY 40202, USA

## Abstract

Recent asthma recommendations advocate the use of long-acting beta-agonists (LABAs) in uncontrolled asthma, but also stress the importance of stepping down this therapy once asthma control has been achieved. The objective of this study was to evaluate downtitration of LABA therapy in pediatric patients who are well-controlled on combination-inhaled corticosteroid (ICS)/LABA therapy. Clinical and physiologic outcomes were studied in children with moderate-to-severe persistent asthma after switching from combination (ICS/LABA) to monotherapy with ICS. Of the 54 patients, 34 (63%) were determined to have stable asthma after the switch, with a mean followup of 10.7 weeks. Twenty (37%) had loss of asthma control leading to addition of leukotriene receptor antagonists, increased ICS, or restarting LABA. There were 2 exacerbations requiring treatment with systemic steroids. In patients with loss of control, there was a statistically significant decline in FEV_1_ (−8% versus −1.9%, *P* = 0.03) and asthma control test (−3.2 versus −0.5, *P* = 0.03). This did not approach significance for FEF_25-75%_, exhaled nitric oxide, lung volumes or airway reactivity. No demographic, asthma control measures, or lung function variables predicted loss of control. Pediatric patients with moderate-to-severe persistent asthma who discontinue LABA therapy have a 37% chance of losing asthma control resulting in augmented maintenance therapies. Recent recommendations of discontinuing LABA therapy as soon as control is achieved should be evaluated in a prospective long-term study.

## 1. Introduction

Since the introduction of long-acting *β*2-agonists (LABAs) in the 1990s and their approval in the United States in 1994, there has been data showing their benefit on lung function and clinical outcomes [1]. These agents are widely used for asthma treatment in combination with inhaled steroids, and may be overused [2, 3]. Reports of increased adverse events in patients receiving this therapy, as noted in the Salmeterol Nationwide Surveillance (SNS) study, led the US Food and Drug Administration (FDA) to request a safety and monitoring trial that started enrollment in 1996 [4]. However, enrollment was stopped in 2003 related to increased asthma-related deaths in patients receiving salmeterol [5]. Despite uncertainty regarding concomitant use of anti-inflammatory therapy in this study, a black box warning for LABAs was introduced. The FDA, after comprehensive reviews and public discussions at multiple advisory meetings in 2005, 2007, and 2008, released new safety requirements for combination therapy in February, 2010 [4]. The FDA recommended that “LABAs should be used for the shortest duration of time required to achieve control of asthma symptoms and discontinued, if possible, once asthma control is achieved” [6].

While there are data showing that for both pediatric and adult patients whose asthma is not well controlled, the addition of LABA therapy is clinically superior to doubling the inhaled corticosteroid dose or the addition of a leukotriene receptor antagonist [7, 8]; there are little data or clinical practice guidelines on how best to step off LABA therapy. Previous studies have shown that the removal of LABA leads to lower lung function and less well-controlled asthma, but there are no pediatric data available [9, 10]. Current asthma guidelines suggest stepping down the dose of inhaled corticosteroids rather than discontinuation of LABA [11]. This leaves clinicians torn between having to choose between FDA and expert opinion in the management of their patients with persistent asthma. Unfortunately, the safety risks will not be clarified until the multinational, randomized, and double-blinded prospective combined FDA and Pharma initiative is completed in about 6 years [12].

This investigator-initiated study was designed to evaluate short-term clinical outcome after the discontinuation of LABA therapy in a population of well-controlled children with asthma.

## 2. Methods

This study included patients with moderate-to-severe persistent asthma on the basis of the criteria recommended by the National Asthma Education and Prevention Program [11], and an ability to perform reproducible spirometry. Patients were excluded if they had concomitant primary pulmonary disease (e.g., cystic fibrosis and primary ciliary dyskinesia). All patients were followed at the Childhood Asthma Care and Education Center at Kosair Children's Hospital, Louisville, Ky, USA, every three to four months. Asthma control was based on criteria [11] that included asthma control test score (ACT) ≥ 20 [13] and normal or near normal spirometric data. Once these criteria were met, the maintenance therapy was switched from combination therapy to ICS monotherapy. The choice of inhaled steroid was made by the pulmonologist after consideration of insurance coverage, patient factors, and physician preference. Once the LABA was discontinued, the patients remained on bioequivalent doses of inhaled steroids [11, 14]. For example, patients on fluticasone/salmeterol combination were switched 1 to 1 (microgram-microgram) to either fluticasone propionate-HFA (FP-HFA), or beclomethasone-HFA (BDP-HFA), mometasone furoate (MF), or ciclesonide (CIC). Patients on budesonide/formoterol combinations were switched 2 to 1 to BDP-HFA, FP-HFA, MF, or CIC. Standardized education was completed by an asthma educator to review device and spacer technique.

Patients were reevaluated after 8 weeks of therapy. Information recorded included symptom control (cough, wheeze, shortness of breath, and nocturnal symptoms), use of rescue medications including oral steroids, spirometry, lung volumes, exhaled nitric oxide, and ACT scoring. Throughout the study, patients using MDI had to use it with a spacer device with inspiratory flow signal (Aerochamber, Monaghan Medical Corporation, Plattsburgh, NY, USA). All patients received education by a respiratory therapist before the start of the study on device technique. Standard spacer technique included slow inhalation with 10 sec breath hold before exhalation and 4–6 regular tidal breaths/activation. All patients understood and adequately reproduced the technique. None of the patients developed hoarseness or oral candidiasis during the study. Patients were instructed to wash and clean their spacer once a week. Atopy was defined by positive allergy skin test or Immunocap *in vitro* quantitative assay, use of immunotherapy, or physician-diagnosed allergic rhinosinusitis.

Compliance was discussed with each patient on each visit. At each visit, patients were required to give the names and the daily doses of all of their asthma medications, including the inhaled steroids. Patients were also asked about their compliance with each medication, including inhaled steroids, and the information was confirmed with the parents.

Patients were considered to be uncontrolled if they met one of the following: systemic steroid use due to asthma exacerbation, drop in FEV_1_ of at least 12% or FEF_25–75%_ of 25%, or a decrease in ACT score to <20. The primary outcome was maintenance of asthma control. Secondary outcomes included the change in FEV_1_, FEF_25–75%_ or ACT score, and the value of exhaled nitric oxide (eNO).

Lung function was assessed at each clinic visit by spirometry which was performed according to the American Thoracic Society Standardization of Spirometry Guidelines (1995) [15].The value recorded for FEV_1_ was the highest of three American Thoracic Society- acceptable curves from three separate tests. The prediction equation of Polgar and Promadhat was used to determine predicted values [16]. All evaluation was done using the same spirometer (Koko Trek spirometer, Ferraris, Louisville, CO, USA), plethysmograph (MedGraphics Elite Series, Medical Graphics Corporation, St. Paul, MN, USA) and nitric oxide analyzer (Niox Mino, Aerocrine, New Providence, NJ, USA). Pneumotachometer used was Model Number 91-000. Calibration was done daily.

All statistical analysis was performed with SPSS, version 19.0.0 (IBM). The Spearman correlation was utilized due to noncontinuous or not normally distributed data. The test used with continuous outcomes between groups was the *t*-test if normally distributed or the Mann Whitney if not normally distributed. The institutional review board approved this retrospective review.

## 3. Results

### 3.1. Patient Characteristics and Medication Use

A summary of select variables pertinent to our analysis is presented in Table 1. All patients were receiving inhaled corticosteroids and long-acting beta-agonist therapy. Eight patients were receiving the fluticasone-salmeterol combination in one single device, and 43 the budesonide-formoterol combination and the remaining 3 ciclesonide and formoterol by separate devices. All of the budesonide-formoterol combinations were delivered by HFA. Three of the fluticasone-salmeterol combinations were delivered by HFA. Patients were enrolled over a nine-month period, from March through December.

Of those on fluticasone-salmeterol, 4 were started on Mometasone, 3 on Beclomethasone, and 1 on fluticasone. Of those on budesonide-formoterol: 21 were started on Mometasone, 18 on Beclomethasone, and 4 on budesonide. The remaining 3 on Ciclesonide had their formoterol discontinued, remaining on Ciclesonide alone.

### 3.2. Asthma Control at Followup

Patients were followed at 10.7 weeks (+/− 5.2, range 1–24). Thirty-four (63%) had maintained asthma control based on symptoms and spirometry in accordance with NHLBI guidelines. The remaining 20 (37%) had loss of asthma control. Of these, 8 had a decline in ACT score to <20 with associated spirometric evidence of increased airflow obstruction. Eight patients had a decrease in ACT score alone, including 2 patients that required a 5-day course of oral prednisone due to an asthma exacerbation. The remaining 8 patients had an increase in airflow obstruction without a decrease of ACT to <20. No patients required hospitalization during the followup period. All patients received bronchodilator testing at followup. Only 1 patient in the uncontrolled group had evidence of airway hyperresponsiveness.

Of the patients who had uncontrolled asthma at followup, 14 were initially receiving the budesonide-formoterol combination. Of these, 8 had been switched to beclomethasone, 4 to mometasone, and 2 remained on budesonide. Of the 3 patients on fluticasone-salmeterol that were uncontrolled, they were switched to beclomethasone, mometasone and fluticasone. All three of the patients receiving ciclesonide-formoterol and switched to ciclesonide alone had uncontrolled asthma at followup.

These findings prompted the restarting of LABA therapy in 18 patients. In the other 2 patients, the ICS was doubled in one due to elevated exhaled nitric oxide and LTRA was started in the other due to known underlying atopy.

**Figure 1:**
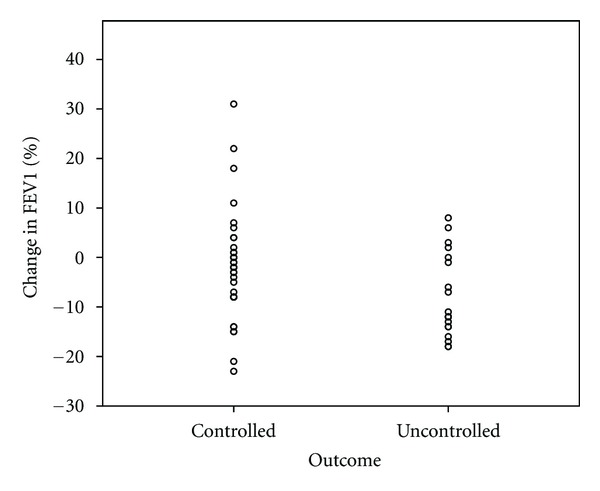
Scatterplot showing patient's change in FEV_1_ (% predicted) comparing those that maintained asthma control off LABA therapy at followup (controlled) and those that experienced loss of asthma control (uncontrolled).

**Figure 2:**
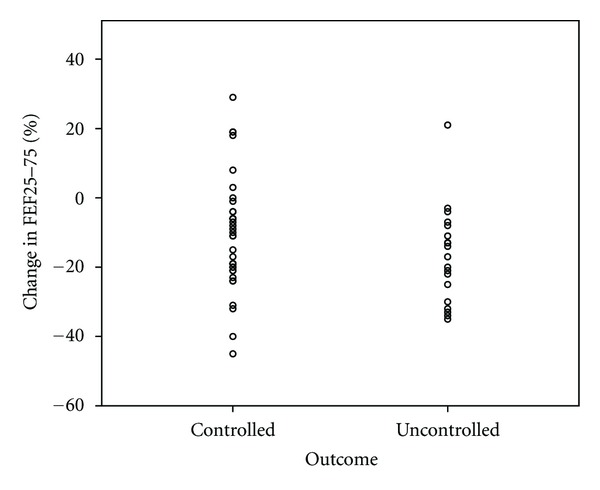
Scatterplot showing patient's change in FEF_25–75%_ (% predicted) comparing those that maintained asthma control off LABA therapy at followup (controlled) and those that experienced loss of asthma control (uncontrolled).

**Figure 3:**
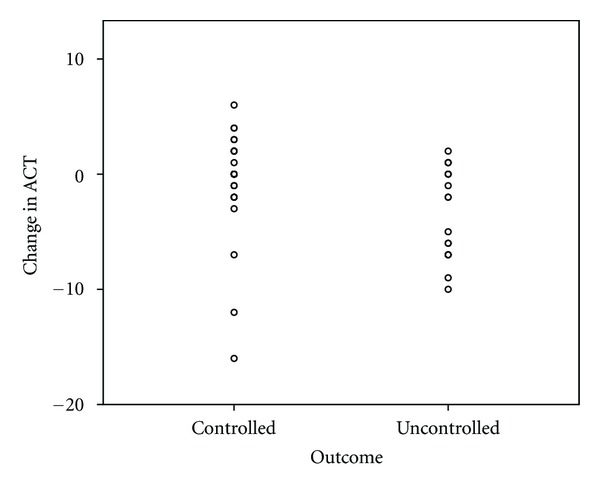
Scatterplot showing patient's change in ACT score comparing those that maintained asthma control off LABA therapy at followup (controlled) and those that experienced loss of asthma control (uncontrolled).

A summary of the between-group differences of those maintaining and losing control is provided in Table 2. There were no differences at baseline that predicted who could successfully have their LABA therapy stopped. This includes which anti-inflammatory therapy the patients were receiving and what steroid moiety they were subsequently changed to. At followup there were significant group differences in terms of symptomatic and spirometric control. Figures 1, 2, and 3 display scatterplots of individual patient's change in lung function and ACT scores. In the controlled, group those with a decrease in lung function had percent predicted values over 100%, normal FEV_1_/FVC, ratio and unchanged ACT. Likewise, those with a decrease in ACT had unchanged spirometric data and were felt to clinically have either a viral upper respiratory illness or allergic rhinosinusitis. Eleven patients in the controlled group and 7 in the uncontrolled group had lung function and exhaled nitric oxide evaluation at followup. There was no significant difference in exhaled nitric oxide or evidence of hyperinflation. However, the uncontrolled group did have a significantly elevated residual volume over total lung capacity ratio (RV/TLC) (0.31 versus 0.22, *P* < 0.05) indicating more air trapping in the group off LABA therapy. No group differences were present based on date of study enrollment.

## 4. Discussion

Asthma guidelines have advocated step-down therapy once asthma is well controlled [11]; however, there is little evidence for how this should be accomplished. With the FDA recommending strongly against the use of LABA therapy [6], this study was designed to evaluate whether pediatric patients with well-controlled persistent asthma on combination ICS/LABA therapy were able to maintain short-term asthma control with the discontinuation of their LABA component. Thirty-seven percent of the patients had loss of asthma control necessitating systemic steroids or augmentation of their baseline controller therapy. The loss of spirometric control in a subset of these patients, without a significant increase in symptoms, points to the importance of obtaining objective measures of pulmonary function in the management of pediatric asthma, whenever a therapy change is contemplated.

This study highlights the challenges faced by clinicians on a daily basis. For patients with uncontrolled asthma, there is evidence regarding the utility of adding LABA therapy. Conversely, there are little data, specifically in pediatric patients, on the discontinuation of LABA therapy. Studies from the early 1990s were the first to illustrate the beneficial effects of adding LABA therapy for patients with uncontrolled asthma [17, 18]. The publication of the Formoterol and Corticosteroids Establishing Therapy (FACET) study in 1997 added to the growing body of evidence and led to widespread use of these agents [19]. This double-blind, placebo-controlled study involving 852 adult asthmatics showed a significant reduction in asthma exacerbations over a 1-year period in those treated with formoterol in addition to ICS. Recent meta-analyses have shown the improvement in clinical control, with decreased clinical symptoms and exacerbations, and improved objective measures of pulmonary function with the use of LABA therapy [20, 21]. The recent publication of the Best Add-on Therapy Giving Effective Responses (BADGER) study extended the role of LABA in uncontrolled pediatric asthma [8].This randomized, double-blind, three-period crossover trial for 48 weeks evaluated the response to increased ICS and the addition of LABA or montelukast in pediatric patients with uncontrolled asthma. The response to LABA step-up therapy was the most likely to provide the best response of the three.

The effectiveness of LABA therapy in the management of asthma has been dampened by safety concerns since the time of their FDA approval. The available data and their interpretation regarding safety implications has been ongoing, culminating in 2010 with the release of specific label changes for LABA and planning for a safety megastudy. The label change included “stop use of LABA, if possible, once asthma control is achieved and maintain use of an asthma-controller medication, such as inhaled corticosteroid” [5].

How best to step off LABA therapy is an open question based on available data. Maintaining LABA while reducing the dose of ICS has been shown to maintain control [22]. Extending the SMART (single maintenance and reliever therapy) use of the combination ICS/formoterol product may be another option [23]. Both options, however, advocate the continued use of a LABA. Studies looking at stopping the LABA component have uniformly shown worsening symptoms or quality of life, increased SABA therapy, or worsened pulmonary function [22]. Unfortunately, these studies did not include a pediatric population.

This first report of the loss of asthma control in the pediatric setting is limited by the lack of a control or comparison group, randomization, and blinding. These are inherent biases in an observational study evaluating a universal change in a clinical practice. However, each patient in effect acted as his/her own control, since their degree of control was compared to themselves before the clinical change. During this time our patient care goals were to stop LABA therapy entirely, so a comparison group of lower dose ICS/LABA combination was not considered. With the group's normal spirometry one could postulate that our patients were overtreated for their asthma. However, that strengthens the findings since control should have been maintained by the decrease in therapy. We were unable to get lung volume and exhaled nitric oxide measurements on all of the patients at followup as desired. Reasons for this included patients being seen for a sick visit, insurance coverage, or parent preference. Nor are those measurements part of our routine practice, so baseline measurements are not available. However, there was a statistically significant increase in RV/TLC ratio in the group that lost control despite the small patient number. With the dose of ICS being stable one would not expect the eNO to change significantly with the discontinuation of LABA.

In a secondary analysis, there was no association of lack of control with race or ethnic group. However, this study was not powered to do so, nor did it include genetic polymorphism analysis. Studies have shown a differential response to asthma therapy based on racial demographic [24, 25]. The Badger trial showed that for LABA therapy, this was the add-on therapy most likely to improve control. Whereas in African Americans subjects, this therapy was shown to be no better than increasing ICS at improving control [8]. And recently, the Asthma Clinical Research Network (ACRN) showed an increased rate of treatment failure in African Americans being treated with LABA therapy regardless of concomitant use of other controller therapy, including ICS [25]. This finding would serve to add credence to the SMART study which showed increased risks in African Americans treated with LABA [5]. These differential responses may be related to socioeconomic or other unknown factors. Perhaps the decision for LABA therapy will be personalized in the future, and based on risk-benefit profile and genetic polymorphisms.

Those eight of our patients who had a significant decline in airflow despite no noticeable worsening of symptoms further reminds us of the role of spirometry in asthma management. The Expert Panel Report 3 (EPR-3) recommends the use of spirometry in the diagnosis and periodic monitoring of patients with asthma [11]. The use of symptoms alone has been shown to underrepresent the degree of asthma severity [26]. Symptoms are fairly reliable indexes of asthma control; however, symptoms and lung function do not always correlate. Some patients who are poorly controlled based on objective measures of lung function perceive few, if any, symptoms. These “poor perceivers of airflow obstruction” are at increased risks of asthma morbidity and mortality [27].

## 5. Conclusion

The goals of most asthma guidelines, as well as the recent FDA concerns, can be met in most patients. However, this study adds to the mounting literature of a group of asthmatics who gain better control on LABA therapy and subsequently lose control when it is discontinued. Clearly, LABA therapy is not acceptable as monotherapy, and the use of combination ICS/LABA therapy in one single device precludes this option. If achieving symptomatic and spirometric control is the goal in pediatric asthma, the availability of LABA therapy as an added option appears necessary. Future prospective trials looking at LABA and stratifying risk by race, gender, and phenotypic response will help further to clarify their role.

## Figures and Tables

**Table 1:** Baseline patient characteristics.

	All
Patients, *n *	54
Age, y	10.9 (6–18)
Male sex (%)	33 (61%)
Caucasian race (%)	44 (81%)
Duration of asthma, yr	7.4 (1–15)
LTRA use (%)	25 (46%)
ACT	23.5 ± 1.9
FVC, % predicted	104.8 ± 11.6
FEV_1_, % predicted	102.3 ± 11.8
FEF_25-75_, % predicted	98.1 ± 24.7

Abbreviations—LTRA: leukotriene receptor antagonists; ACT: asthma control test; FVC: forced vital capacity; FEV_1_: forced expiratory volume in 1 second; FEF_25-75_: forced expiratory flow at 25–75% of FVC.

**Table 2:** Patient characteristics comparing those that maintained asthma control off LABA therapy at followup (controlled) and those that experienced loss of asthma control (uncontrolled). ^∗^represents *P* < 0.05.

	Controlled	Uncontrolled
Patients, *n *	34	20
Time to f/u, weeks	10.3	11.5
Age, yr	11.3	10.2
Male sex (%)	22 (65)	11 (55)
Caucasian (%)	27 (79)	16 (80)
Insurance, government (%)	7 (21)	8 (40)
Smoke exposure, negative (%)	19 (56)	14 (70)
Atopy (%)	27 (79)	15 (75)
Duration of asthma, yr	7.6	6.4
LTRA use (%)	15 (44)	10 (50)
ACT, baseline	23.5	23.4
FVC, baseline % pred	105 ± 11.6	104 ± 12.1
FEV_1_, baseline % pred	102 ± 11.8	100 ± 10.5
FEF_25-75_, baseline	101 ± 22.2	93 ± 28.7
Change ACT	−0.5 ± 4.1	−3.2 ± 3.9*
Change FEV_1_, % pred	−1.9 ± 11.0	−8.0 ± 8.5*
Change FEF_25-75_, % pred	−10.8 ± 16.4	−16.9 ± 13.8
TLC, %	105.9 ± 22.0	100.4 ± 11.2
RV/TLC	0.22 ± 0.07	0.31 ± 0.09*
eNO	23.8 ± 25.6	26.7 ± 25.8
Exacerbation	0	2
Hospitalization	0	0
